# Local economic inequality and mortality: Too little within-unit variation for fixed effects analysis?

**DOI:** 10.1177/14034948241259914

**Published:** 2024-08-07

**Authors:** Jon Ivar Elstad, Kristian Heggebø, Espen Dahl

**Affiliations:** 1 NOVA, Centre for Welfare and Labour Research, Oslo Metropolitan University, Norway; 2 Faculty of Social Sciences, Oslo Metropolitan University, Norway

**Keywords:** Fixed effects analysis, within-unit variation

## Abstract

This debate article discusses the use of fixed effects models for causal analysis, with reference to an article recently published in *Scandinavian Journal of Public Health*.

Jørgensen and Lyngstad’s article [1] ‘Does local income and wealth inequality affect mortality?’, published in *Scandinavian Journal of Public Health*, 52(1): 58-63, is a recent addition to Nordic studies [2–9] on the so-called Income Inequality Hypothesis. This hypothesis states that higher income inequality, net of other health determinants, impacts negatively on population health. Using register data, Jørgensen and Lyngstad (hereafter J&L) calculated annual Gini coefficients for income and wealth in all Norwegian municipalities across 1993–2013. Cross-sectional findings were in line with the hypothesis, as municipalities with higher income inequality had higher mortality (see p.62 and Supplemental material Table S7 online). Similar, but weaker, associations appeared for wealth inequalities. However, due to possible omitted-variable bias, cross-sectional results cannot ascertain a *causal* effect of municipal economic inequality. J&L proceeded therefore with fixed effects analyses that control for all time-invariant municipal circumstances, even when measurements are lacking [10,11]. In their fixed effects results, practically all associations between municipal economic inequality and inhabitants’ mortality disappeared, and the authors concluded that there is ‘no evidence of an association’ [1: p.62].

J&L’s study is laudable in several ways (for instance addressing not only income, but also wealth inequality), but there are debatable aspects. In this commentary, we will raise a question about their fixed effects analyses. The distinguishing feature of this method is that possible causal effects are estimated solely from *within-unit variations*, while *between-unit differences* are ignored. This approach requires, however, a reasonable amount of intra-municipal changes in economic inequality, cf. textbooks: ‘the predictor variables of interest [here: municipal economic inequality] must change in value (on) multiple occasions for some substantial portion of the sample’ [10: p.2]. Without ‘sufficient variability over time in the predictor variables’ [11: p.370], results can be very imprecise.

J&L do not discuss to what extent this requirement was satisfied in their data. In order to probe into this issue, we have used the same Norwegian register data (available for researchers at microdata.no/en/) and calculated municipal *income* Gini coefficients in exactly the same way as J&L, that is, annually for the 424 municipalities that existed practically unchanged 1993–2013, based on pensionable incomes for male inhabitants aged 30–69 years.

Findings are reported in Table I. *Between-municipal* differences in income inequality seem substantial. The average municipal Gini across 1993–2013 was 0.389, while 110 of the 424 municipalities had an average below 0.365, and 90 municipalities had average Ginis at least 0.050 higher, that is, above 0.415. But were *within-municipal* variations substantial?

**Table I.** Variations in Gini income inequality in Norwegian municipalities 1993–2013.

	All	Largest fourth	Next to largest	Next to smallest	Smallest fourth
**Between-municipal variation**					
Number municipalities	424	106	106	106	106
Appr. % of population	100.0%	74.0%	15.1%	7.5%	3.4%
Average municipal Gini across 1993–2013 (SD)	0.389 (0.035)	0.380 (0.025)	0.377 (0.030)	0.394 (0.033)	0.403 (0.042)
Distribution					
Below 0.365. No munic.	110	34	37	19	20
Mean Gini	0.348	0.352	0.345	0.350	0.343
0.365 to 0.415. No munic.	224	64	54	62	44
Mean Gini	0.388	0.389	0.385	0.389	0.390
Above 0.415. No munic.	90	8	15	25	42
Mean Gini	0.439	0.426	0.428	0.439	0.446
**Within-municipal variation**					
Number of observations	8904	2226	2226	2226	2226
Average deviation from municipal average (SD)	0.017 (0.015)	0.017 (0.011)	0.017 (0.013)	0.018 (0.015)	0.018 (0.020)
Distribution of deviations Number (%)					
Less than +/- 0.020	4764 (53.5)	1294 (58.1)	1246 (56.0)	1157 (52.0)	1067 (47.9)
+/- 0.020 to 0.035	3228 (36.3)	846 (38.0)	833 (37.4)	824 (37.0)	725 (32.6)
+/- 0.035 to 0.050	779 (8.7)	84 (3.8)	141 (6.3)	219 (9.8)	335 (15.0)
More than +/- 0.050	133 (1.5)	2 (0.1)	6 (0.3)	26 (1.2)	99 (4.4)

In each of the 424 municipalities, there were 21 deviations from the municipality’s average during 1993–2013, making up a total of 8904 within-municipality deviations. Table I shows that 53.5% of them were less than plus/minus 0.020. Another 36.3% were in the plus/minus 0.020 to 0.035 range – changes that we doubt would be capable of making much difference for the inhabitants in the short run. Perhaps Gini increases of more than 0.050 – or decreases of more than 0.050 – would have a noteworthy impact? But such deviations occurred only 133 times (1.5% of the 8904 deviations). Among them, only two (!) took place in the 106 largest municipalities (with about 74% of the entire population), while 99 occurred in the smallest quarter of the municipalities, where less than 4% of the population lived.

What constitutes ‘noteworthy’ change is of course arguable. One benchmark might be the between-municipal variations which J&L showed had statistical significance. Average Gini was 0.348 in the 110 municipalities (out of 424) at the lower end of the inequality scale, and about 0.09 higher (0.439) in the 90 municipalities in the upper end of that scale (Table I). Table I indicates clearly that within-municipal variations were much smaller.

Another reference could be the well-known Wilkinson/Pickett-thesis [12] that higher income inequalities erode social cohesion and social solidarity, thereby inflicting psychosocial stress and more ill health. Given that this thesis is correct: how much would municipal income inequality have to change in order to affect population health? It seems unlikely that a Gini increase of 0.01, or 0.02, or even 0.03, could generate, in a relatively short time period, such alterations in social life that population health would deteriorate. And is it likely that a *decline* in income inequality, say, from 0.38 to 0.34, would provoke a significant drop in mortality from one year to the next? When arguing for their thesis, Wilkinson/Pickett pointed to rich countries and US states where typical differences between Gini coefficients were much larger.

To us, Table I suggests that the within-variations in municipal income inequality in J&L’s data were too small for applying fixed effects models. In other words: their findings cannot be considered an empirically substantiated rejection of the Wilkinson/Pickett thesis. The general point, we believe, is that one should observe a set of important recommendations when fixed effects analyses are used: ‘Researchers should consider the degree of variation in their focal variables. Is there enough variation to generate reliable coefficient estimates? Or are the coefficient estimates . . . really an artifact of insufficient variation?’ [13: p.366].
